# Ammonia and coma – a case report of late onset hemizygous ornithine carbamyltransferase deficiency in 68-year-old female

**DOI:** 10.1186/s12883-020-01700-9

**Published:** 2020-04-06

**Authors:** Justus Marquetand, Peter Freisinger, Tobias Lindig, Sebastian Euler, Michael Gasser, Dietrich Overkamp

**Affiliations:** 1 Center of Neurology, Hertie-Institute for Clinical Brain Research, University of Tübingen, Tübingen, Germany; 2 Department of Consultation Psychiatry and Psychosomatic Medicine, University Hospital Zürich, Zürich, Switzerland; 3 Department of Pediatrics, Klinikum Reutlingen, Reutlingen, Germany; 4 Department of Neuroradiology, University of Tübingen, Tübingen, Germany; 5 Department of Neurology, Kliniken Calw-Nagold, Calw, Germany; 6 Department of Internal Medicine, University of Tübingen, Tübingen, Germany

**Keywords:** Hemizygous ornithine carbamyltransferase deficiency, OTC, Ammoniac, Coma, Late onset

## Abstract

**Background:**

Acute hyperammonemia without signs of common causes in the elderly might be challenging to identify. We report the oldest case known to date of a female patient with late onset ornithine carbamyltransferase deficiency (OTC), which was unmasked after a protein overload due to nutritional supplements. Our case illustrates how environmental factors (protein overload) in previously unknown OTC in the elderly leads to hyperammonemic encephalopathy and highlights that early treatment prevents persisting neurological deficits and should be considered in absence of common causes of hyperammonemic encephalopathy.

**Case presentation:**

A 68-year-old woman presented with acute confusion, which progressed into a deep coma (Glasgow-Coma-Scale score 3) within a few hours.

The only remarkable finding was a plasma ammonia (NH3) concentration of 697 μmmol/l (range 12–47 μmmol/). Third party history revealed that the patient disliked meat for most of her life (meat = protein, which needs to be metabolized) and had taken nutritional supplements (since supplements often have a high protein-ratio) 2 days before the symptoms started. Protein catabolism results in NH3, which is metabolized via the urea cycle. Consequently, the acute hyperammonemia in our patient was thought to be related to an inherited metabolic disorder, which only unmasked itself as a result of an overload of the corresponding metabolite (in this case protein). Since ornithine carbamyltransferase deficiency (OTC) is the most common inherited urea cycle disorder, this diagnosis became likely and was confirmed later via genetic and metabolic testing (amino acids, orotic acid, etc.). After 2 weeks of treatment (dialysis, low-protein-diet, nitrogen-lowering medication) the patient was discharged in a healthy condition without any neurological deficits.

**Conclusion:**

OTC is a x-chromosomal linked disorder, that usually manifests in newborn infants and children, but also rarely in adults and even rarer in the elderly (50- till 60-years-old), where it is probably underdiagnosed. In case of hyperammonemic encephalopathy – regardless of the underlying cause -, treatment should be started early to prevent persisting neurological deficits. OTC should be considered in absence of common causes of hyperammonemic encephalopathy.

## Background

Elevated NH3 levels in patients are not uncommon and often correlated with mild to severe encephalopathy. Next to common causes like liver or kidney diseases, hereditary disorders of the urea cycle can also lead to acute hyperammonemia. Acute hyperammonemia as the only remarkable finding without evidence of more common causes might be challenging for many clinicians. In such situations, it is important to focus on the pathophysiology of hyperammonemia. NH3 accumulates due to protein catabolism and is metabolized via the urea cycle. Consequently, acute hyperammonemia can be related to a disorder of the urea cycle, e.g. inherited disorders of the urea cycle. Inherited disorders of the urea cycle decompensate whenever a protein breakdown (dietary proteins or fasting) and NH3 accumulation exceed detoxification capacity of the urea cycle. The most inherited urea cycle disorder is the x-chromosomal linked ornithine carbamyltransferase deficiency (OTC), which usually manifests in newborn infants and children. OTC is rare in adults and even rarer in the elderly (50- till 60-years-old), where it is probably underdiagnosed. One reason for underdiagnosis might be a broad variety of initial symptoms due to hyperammonemic encephalopathy [1] and clinicians being unaware of OTC [1]. The exact pathophysiological mechanism around why and how OTC manifests at advanced age remains elusive, but cases with OTC manifesting in the elderly might point out the potential role of environmental and other genetic factors on disease expression. Our case of a 68-year-old woman with late-onset OTC (to date the oldest case of a female with late-onset OTC [2]) emphasizes that every clinician should be aware of late-onset inherited metabolic disorders and illustrates how environmental factors might trigger hyperammonemic encephalopathy.

## Case presentation

A 68-year-old woman (height 168 cm, body weight 60 kg, BMI 21,3 kg/m^2^) presented with acute confusion, which progressed into a deep coma (Glasgow-Coma-Scale score 3) within a few hours. Immediate admission to the local hospital was initiated, where intubations and mechanical ventilation were started. A preliminary diagnostic workup revealed no pathological findings in cranial CT-scans (no edema, bleeding, etc.), CSF-studies and laboratory investigations,

- except for a plasma ammonia (NH3) concentration of 697 μmmol/l (range 12–47 μmmol/).

**Fig. 1:**
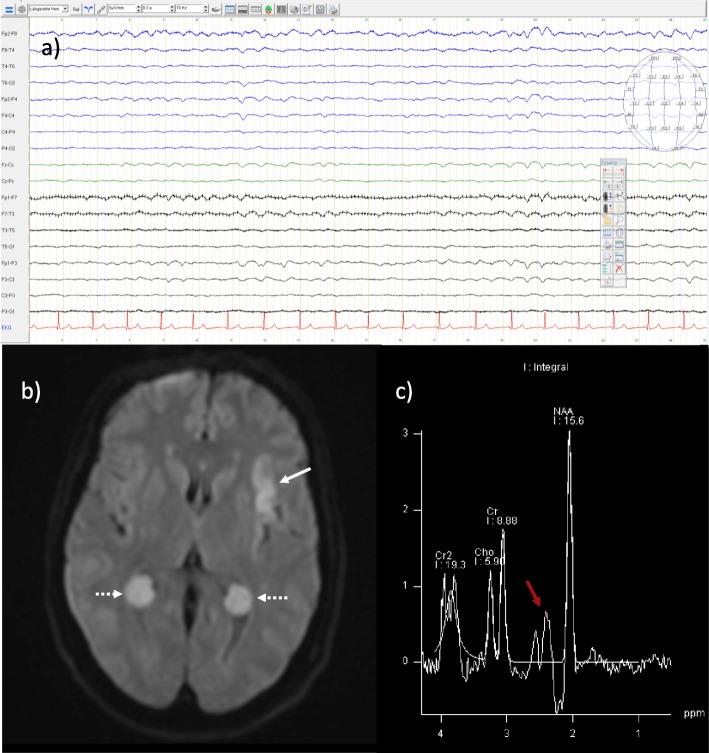
Diagnostic findings: **a** EEG showed frontal intermittent rhythmic delta activity (FIRDA) and a low-voltage amplitude. No sedative medication was administered 3days before or during the recording of the EEG. **b** Diffusion weighted imaging (DWI) in MRI showed a hyperintense signal in the left insula (white arrow), an incidental finding of benign antenatal plexus choroid cysts (dashed arrows) and single-voxel MRI-spectroscopy (NAA: N-acetylasparate; Cr: creatinie; Cho: choline; Cr2: Creatine peak 2) **c** an increase of glutamine and decrease of myoinositol and choline

**Fig. 2:**
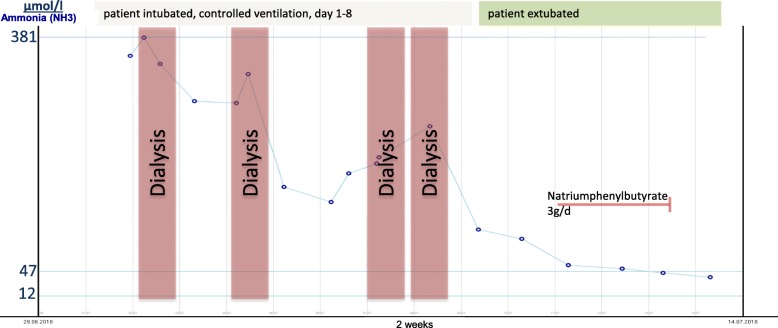
Ammonia (NH3) levels throughout the course of 2 weeks. Four dialysis-sessions took place, which led to a corresponding decrease of plasma NH3 concentration. Between day 7 and 8 there was an increase of NH3, which required another dialysis session. One day later, the patient regained consciousness and could be extubated

During the first 5 days, EEG, MRI, abdominal ultrasound and extensive laboratory-studies were conducted (see Fig. 1). After 2 weeks of treatment (Fig. 2), the patient was discharged in a healthy condition without any neurological deficits.

The disease underlying the patient’s condition remained mysterious. The only significant information was the medical history by third party (the husband), which revealed that the patient had started to consume protein-based nutrition supplements 2 days prior to admission. The patient had unintentionally avoided significant meat consumption most of her life, most notably during the last five to ten years.

Consequently, acute hyperammonemia can be related to primary disorder of the urea cycle, e.g. inherited disorders of the urea cycle, or due to a secondary inhibition of the urea cycle (organics acidurias or fatty acid oxidation defects).

## Discussion and conclusion

Acute hyperammonemic encephalopathy due to hemizygous ornithine carbamyltransferase deficiency (OTC) is rare in adults, but – as illustrated by this case – may lead to serious and even life threatening conditions. Once identified, it is a treatable condition with the potential of complete recovery.

How to identify this rare, but treatable genetic disorder? - Since common causes of hyperammonemic encephalopathy were not apparent, a more pathophysiological approach was required. Third party history revealed that the patient disliked meat for most of her life (meat = protein, which needs to be metabolized) and took nutritional supplements (possible protein overload, since supplements often have a high protein-ratio) 2 days before the symptoms started. Consequently, an inherited metabolic disorder, which only unmasked itself as a result of an overload of the corresponding metabolite (in this case protein), was identified as a probable cause. Since OTC is the most common inherited urea cycle disorder [2], this diagnosis became likely.

Which tests are needed? – Metabolic and genetic testing. We suggest using the international guidelines on urea cycle disorders [3], since the profile of specific amino acids might suggest a disorder of the urea cycle. Metabolic testing showed increased uracil (134 mmol/molCreatinine, range 0–29 mmol/molCreatinine) ornithine (275 μmol/l, range 36–96 μmol/l), orotic acid values (2,88 mmol/molCreatinine, range 0–1.47 mmol/molCreatinine), whereas citrulline showed normal values (8.5 mmol/molCreatinine, range 0–19.3 mmol/molCreatinine). Also in plasma citrulline levels were normal, but glutamine was increased (1067 μmol/l, range 340–740 μmol/l). Additonally an acylcarnitine profile was performed via blood spot test, which ruled out a fatty oxidation and organics acidurias leading to secondary urea cycle defects. Genetic testing serves as conformational evidence for the diagnosis and helps in case of counseling in family planning or for relatives. Genetic testing (panel diagnostic for urea cycle disorders) showed a hemizygous mutation in c.995G > A; p.Trp332*.

OTC is a x-chromosomal linked disorder, that usually manifests in newborn infants and children, but also rarely in adults and even more rarely in the elderly (50- till 60-years-old), where it is probably underdiagnosed [1]. In the newborn infants and children, symptoms seem to be more homogenous. Especially males initially present with irritability, lethargy and feeding problems, which progress into coma or seizures [2]. Adults show a more variable phenotype, which ranges from subtle psychiatric manifestations to coma, often associated with previous factors of increased catabolic stress (e.g., trauma, nutrition, medications like sodium valproate, intoxication, etc.) [4]. Why and why not OTC manifests in later life, remains elusive, but in our case also lyonization of the non-mutated x-chromosome might be relevant.

Treatment depends on symptom severity. In severe cases (e.g., coma) acute treatment consists of dialysis, administration of nitrogen-eliminating drugs like phenylacetate/−butyrate or benzoate and low-protein diet, which can lead to full recovery [5]. If not treated at an early stage, neurological deterioration continues and the risk of death is imminent [6]. To our knowledge, we describe here the case of the oldest female with late-onset-OTC. Since OTC is a x-chromosomal linked disorder of the urea cycle, and approximately only 20% of female carriers of the OTC gene become symptomatic [2], our case appears to be of even more practical interest.

After fourteen days in our hospital, the patient was discharged without any neurological deficits. She continued her low-protein-diet, but no further nitrogen-lowering medication (natriumphenylbutyrate 3 g/d) or dialysis were necessary.

## Learning points

- In case of hyperammonemic encephalopathy – regardless of the underlying cause - treatment should be started early to prevent persistenting neurological deficits. Performing metabolic plasma and urine tests could lead to a fast diagnosis, especially in hyperammonemia without common causes.
- OTC should be considered in absence of evidence for more common causes of hyperammonemic encephalopathy.

## Data Availability

I, Justus Marquetand, the Corresponding Author of this article contained within the original manuscript which includes any diagrams & photographs within and any related or stand alone film submitted (the Contribution) has the right to grant on behalf of all authors and does grant on behalf of all authors, a licence to the BMJ Publishing Group Ltd. and its licencees, to permit this Contribution (if accepted) to be published in the BMJ Neurology and any other BMJ Group products and to exploit all subsidiary rights, as set out in our licence set out at: http://www.bmj.com/about-bmj/resources-authors/forms-policies-and-checklists/copyright-open-access-and-permission-reuse

## Abbreviations

CSF: Cerebrospinal fluid
CT: Computertomography
DWI: Diffusion weighted imaging
EEG: ELECTROENCEPHALOGRAPHY
FIRDA: Frontal intermittend rhythmic delta activity
MRI: Magnetic resonance imaging
OTC: Hemizygous ornithine carbamyltransferase deficiency
